# Explorer Naturalists

**DOI:** 10.1371/journal.pbio.0030161

**Published:** 2005-05-17

**Authors:** Fernando E Vega

## Abstract

Nancy Pick's recent book *The Rarest of the Rare: Stories behind the Treasures at the Harvard Museum of Natural History* brings a vast collection to life.

**Figure:**
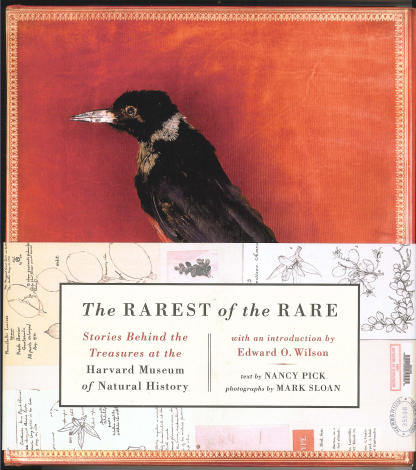


The famous Russian writer Vladimir Nabokov once described writing as “a torture and a pastime” and contrasted it to “a long and exciting career as an obscure curator of lepidoptera in a great museum” [1]. For six years before moving to Cornell University as a professor of Russian literature, Nabokov worked as a research fellow at Harvard's Museum of Comparative Zoology, where he specialized in “blues” (Family Lycaenidae). The public face of this museum (as well as the Harvard University Herbaria and the Mineralogical and Geological Museum) is Harvard's Museum of Natural History, whose collection consists of more than 21 million specimens acquired over two centuries. In a fascinating new book, *The Rarest of the Rare: Stories behind the Treasures at the Harvard Museum of Natural History*, Nancy Pick describes highlights of this vast collection in a rarely found combination of seamless prose, outstanding photographs, history, legendary figures, and, of course, science.

Choosing among 21 million specimens appears a daunting task, and stripped of their historical significance, some of Pick's choices may seem, at first glance, somewhat pedestrian. It is not until we read her description of the specimen of a common sand dollar, that we learn it was collected by Charles Darwin in 1834 during his journeys as a naturalist on the Beagle. Darwin sent the specimen to an echinoid specialist in Switzerland named Louis Agassiz, who later moved to Harvard and obtained funding for the Museum of Natural History, which opened in 1859. A contemporary of Agassiz at Harvard was the famous botanist Asa Gray, who, in contrast to Agassiz, was a strong believer in evolution. One of the items in the collection is an 1857 letter from Darwin to Gray (a photograph of the letter is shown in the book) describing his thoughts on natural selection, two years before *The Origin of Species* was published. Another specimen that's uninteresting until we know its provenance is a turtle that Harvard College graduate Henry David Thoreau sent to Agassiz in 1847. The turtle was collected in the Walden Pond of Thoreau's classic book.

The birds of American history are well represented by a pair of pheasants that the Marquis de Lafayette sent to George Washington in 1786. The original home of these pheasants was in the first scientific museum in the country, founded by the painter Charles Wilson Peale in Philadelphia. When the museum closed in 1849, the specimens eventually found their way to Harvard. A more exotic specimen in the collection, the now extinct bird known as the common mamo (Drepanis pacifica), was collected by Captain James Cook in 1778 in Hawaii. The yellow feathers from this species were used for King Kamehameha's cloak, requiring over 80,000 mamos. In the book, we can also appreciate parts of a dodo skeleton, and an egg from the elephant bird (Aepyornis maximus), a now extinct flightless bird from Madagascar that grew to ten feet tall and laid the largest eggs known for a bird.

In the realm of insects, Pick has chosen to show us some fabulous specimens, including a 35-million-year-old fossil butterfly; the largest insect wing on record, belonging to a dragonfly-like creature collected in Oklahoma and having a wing span of 2.5 feet; and an unbelievable gynandromorphic morpho butterfly, with the wing colors and size of a male on one side, and those of a female on the other.

Pick also presents cases of murder and fraud, including the famous case of John W. Webster, a Professor of Chemistry at Harvard Medical College, who murdered George Parkman. The tale involves Webster's mineral collection and the purchase of a mastodon for the museum. Bearing witness to fraud in science is a painting by John James Audubon in the museum collection. In trying to prove he had depicted the common grouse (Bonasa umbellus) before his competitor, Alexander Wilson (known as the father of American ornithology), Audubon dated his chalk and watercolor illustration with the year 1805. The problem is that the watermark in the paper is dated 1810.

Perhaps the best known specimens at Harvard's Museum of Natural History are the Blaschka Glass Models of Plants, created by Leopold and Rudolf Blaschka in Germany. Originally commissioned for teaching botany by the first director of Harvard's Botanical Museum, an exclusive ten-year contract signed in 1890 extended into 50 years of work and resulted in 4,400 models so exquisite and realistic, it is hard to believe they are actually made of glass.

Other specimens presented in the book include the all-too-familiar regal lily (Lilium regale) collected in China and brought to the United States by Ernest H. Wilson in 1911; a trilobite collected in 1824 by Charles D. Wolcott, discoverer of the famous Burgess Shale fossil deposit in the Canadian Rocky Mountains; a female anglerfish (Linophryne bicornis) showing the parasitic male still attached; a woodpecker (Melanerpes lewis) collected by Meriwether Lewis and believed to be the only complete specimen from the Lewis and Clark expedition (1804–1806); and Richard Evan Schultes's hallucinogenic mushrooms.

*The Rarest of the Rare* brings forward a vivid image of the romance of an era when a large part of scientific research was conducted by “explorer naturalists,” a breed that has become nearly extinct, pushed aside by DNA sequencers, or should we say, “the barcoders of life.” It is hard to put this book away without thinking about all that remains to be discovered in the natural world—the organisms within organisms, the fossils, the remaining new species—because, fortunately, one thing is certain: there is no end to science.
